# What Chinese Women Seek in Mental Health Apps: Insights from Analyzing *Xiaohongshu* User Posts during the COVID-19 Pandemic

**DOI:** 10.3390/healthcare12131297

**Published:** 2024-06-28

**Authors:** Zhenzhen Qin, Sandy Ng, Wenqing Wu, Suxin Zhang

**Affiliations:** 1 School of Design, The Hong Kong Polytechnic University, Hong Kong, China; zhenzhen.qin@polyu.edu.hk; 2 School of Journalism and Communication, Anhui Normal University, Wuhu 241000, China; wuwenqing@ahnu.edu.cn; 3 School of Civil Engineering and Architecture, Hainan University, Haikou 570228, China; sx.zhang@hainanu.edu.cn

**Keywords:** COVID-19 pandemic, female healthcare, therapeutic functionality, female user experience, Xiaohongshu

## Abstract

Gender disparity poses a prominent obstacle to achieving effective mental health outcomes in digital healthcare. Despite women being more inclined to use mental health apps and seeking designs tailored to their specific needs, there is limited research on the factors influencing female users’ engagement with these apps. The COVID-19 pandemic has further exacerbated its disproportionate impact on women’s mental health. This study investigates female users’ posts (n = 5538) about mental health apps during the pandemic, using data collected via a Python web crawler from Xiaohongshu, a popular female-centric social media platform in China. A mixed-methods approach used qualitative thematic analysis and quantitative descriptive statistics. Among these posts, therapeutic functionality emerged as the highest priority, followed by credibility and user experience, with specific design elements highlighted as particularly significant. These findings provide valuable insights for mental health researchers and developers, including you, aiming to create gender-tailored mobile solutions to address the mental health challenges faced by women, especially during future pandemics.

## 1. Introduction

Gender-tailored design features lack detailed elaborations in research related to mental health applications (apps), which offer a range of tools and functionalities to address various psychological disorders [1,2]. Despite the proven efficacy of these apps [3,4], exploring gender-tailored design elements is crucial for inclusive and effective mental healthcare. This is especially important, given the increasing adoption of design approaches integrating healthcare therapies within health apps to address users’ underlying needs [5,6], and the observed greater inclination of females towards accepting mental health apps than males in studies conducted in the United States [7,8]. Moreover, recent studies have indicated that female users prefer particular design features in health apps, such as social support [9]. Additionally, empirical evidence from China has emphasized the importance of tailored mental and physical health apps to address the specific needs of different genders [10]. Therefore, the present study adopts a gender-centric perspective to advance the comprehension of methods and strategies in mental health app design, which is critical in catering to the distinctive requirements of female users and ultimately enhancing their mental well-being.

The COVID-19 pandemic has sparked widespread mental health concerns across the globe [11,12], resulting in increased reliance on mental health apps [13]. Countries like China implemented lockdown policies [14] and showed heightened levels of depression and anxiety across professions [15]. While the pandemic has affected everyone, women have been hit particularly hard emotionally [16]. Research during the early stages of the outbreak in China found that women reported higher levels of mental health issues than men [17]. Factors include motherhood-related challenges like pregnancy and postpartum mental health problems, increased family responsibilities, and rising domestic violence against women, leading to depression and other mental health issues [18]. Women with mental health concerns increasingly rely on mobile apps due to limited access to hospitals and mental health services, especially in China [10]. A study carried out in the United States has found a heightened prevalence of mental health symptoms, particularly in women, when using mental health apps during the COVID-19 pandemic [19]. In this context, exploring women’s voices can offer insights for designing gender-tailored mental healthcare apps.

Recent studies [9,10] have reported a significant need for more focus on the influence of gender-tailored needs in the design of mobile healthcare. This lack may affect the efficacy and uptake of mental health apps among female users since the observed high levels of drop-outs/attrition [20]. Furthermore, previous research on the end-user’s voice of mental health apps has mainly relied on app store user reviews and app ratings regarding attitudes, efficacy, and user experience [20,21,22,23]. However, given their limited expressive functions and convenience, app store reviews may fail to comprehensively understand mental health apps, particularly in addressing gender-tailored needs.

Xiaohongshu, also known as Little Red Book, a female-centric social media platform in China, has become a valuable source of user-generated content [24]. With 90.41% of its active users being female and 83.31% aged between 18 and 34 [25], Xiaohongshu has been examined by scholars for insights into female health concerns, such as postpartum recovery [26] and medical treatment [24]. Analyzing the posts by female users on Xiaohongshu provides crucial theoretical insights into the specific factors and needs surrounding mental health apps during the pandemic. This analysis not only enhances the understanding of mental health app needs for Chinese women but also contributes to the broader discourse on mental health challenges during a pandemic.

Using a Python web crawler, our study examined 5538 posts by female users on Xiaohongshu themed around mental health apps during COVID-19 (from 1 December 2019 to 1 December 2022). It contributes to the existing literature in three ways: firstly, by offering gender-tailored considerations to improve mental health app design for female users; secondly, by emphasizing the importance of data collected during the COVID-19 pandemic for mental health app development; and lastly, by utilizing data from a widely used female-centric social media platform in China to enhance user research through user-generated content analysis.

## 2. Literature Review

### 2.1. Gender Disparities in Adoption of Mental Health Apps during COVID-19

Despite growing recognition of gender-based disparities in the utilization of online healthcare services [9,27], there is a notable lack of research explicitly examining female users’ attitudes and concerns regarding mental health apps, especially during the COVID-19 pandemic, which led to greater adoption of online mental healthcare among female users [10]. Insights from user reviews on mobile stores highlight general opinions on mental health apps, emphasizing user experience, usability, and functionality [20]. Bao and Lee [28] also reviewed the theoretical frameworks and antecedents of health apps in the extensive literature on mobile health. However, these studies do not address gender disparities in depth.

Investigations conducted in countries like Chile [29], Italy [30], and China [31] have reported worsened mental health conditions during COVID-19, with women experiencing significant mental health challenges such as fear and anxiety. While some studies [32] have examined the downloads and activities of mental health apps during COVID-19, they often overlook the gender factor and lack in-depth insights into female users’ experiences.

Research has documented that user experience is crucial for adopting mental health apps, focusing on user interface design [20]. Moreover, studies on fitness health apps indicate that female users prefer specific design features, such as social support [9]. This highlights the need for a comprehensive investigation into female users’ preferences for mental health apps.

The credibility of mental health apps is another critical factor in China, particularly regarding professional development standards during COVID-19 [33]. Credibility encompasses the degree to which mental health apps can be deemed trustworthy and dependable in providing precise, evidence-based, and productive assistance for mental well-being. Recent work on the credibility of mobile services has shown gender disparities; for instance, female users’ perceived credibility of mobile health apps is associated with concern, character, and competence [34]. Other mobile health services, such as pregnancy apps during COVID-19, showed inadequately credible designs [35]. However, specific concerns about the credibility of mental health apps remain unexplored among female users.

Therefore, further investigation is needed to gain a nuanced understanding of female users’ perspectives on mental health apps. To address this gap, we propose the following research question:

RQ1: What dominant factors do female users discuss in their Xiaohongshu posts about mental health apps during COVID-19?

### 2.2. Gender Disparities in Therapeutic Functions of Mental Health Apps during COVID-19

The COVID-19 quarantine, along with social and physical distancing restrictions and the lack of in-person care, led to a surge in the use of mental health apps, mainly for managing anxiety and depression [36]. Evidence suggests that during the pandemic, female users of these health apps experienced higher levels of stress [37], highlighting the need for more efficient and tailored functions for them [38].

Functional designs in mental health apps draw upon various theoretical foundations in medical literature, influencing users differently regarding therapeutic effects and engagement [20]. Therapeutic functions in these apps encompass self-care and social support, which can be categorized into psychological counseling (21.5%), assessment (29.1%), stress relief (7.0%), psychoeducation (14.0%), and multipurpose (28.4%), as observed in China [10]. Additionally, the emergence of artificial intelligence and chatbots facilitates the development of intelligent therapy within mental health apps [39], presenting new possibilities for treating and managing mental health conditions [40].

Self-care behaviors are crucial for promoting mental and physical health [41]. Behavioral change techniques (BCT) are used in mental health apps to encourage self-care, involving observable, replicable components designed to alter or redirect causal processes that manage behavior [42,43]. Cognitive behavioral therapy (CBT), an approach in mental health apps, helps individuals identify and change negative thought and behavior patterns. Features like mobile journaling, meditation, and mood tracking, based on CBT principles, can reduce mental health symptoms [23,44]. Interventions such as journaling and mood tracking correspond to written emotional disclosure (WED), promoting emotional processing by assisting individuals in exposing their emotions for mental health benefits [45,46]. Though survey evidence shows that women experienced higher levels of anxiety and depression symptoms and tended to access self-care recommendations during COVID-19 [47], few studies provide a comprehensive picture of the available self-care functions.

While integrating self-care components into mental health apps is widely endorsed, it is imperative to implement gender-tailored designs to maximize their effectiveness. Women demonstrate higher emotional capabilities, including emotional disclosure and management [48,49], which can impact the efficacy of therapeutic tools such as CBT and WED. Recent evidence suggests that emotional disclosure tools in mental health apps lead to more significant improvements in female users due to their heightened ability to detect changes in emotions [50]. Incorporating additional factors such as social support, self-confidence, interpersonal functioning, and emotion regulation in female-specific CBT can be beneficial for treating clinical conditions like alcohol use disorder [51].

Social support, a counterpart to self-care, is recognized as a crucial strategy for seeking external interaction and has evolved since the 1970s [52]. It is widely discussed in health treatments and is particularly significant for female-specific health issues, including psychological conditions involving emotional challenges [53]. During the COVID-19 pandemic, social support was observed as a protective factor for mental health, including pregnant women [54]. Due to their complex social networks, caregiving responsibilities, experiences of discrimination, and susceptibility to mental health issues, social support can be beneficial for promoting the well-being of women. Incorporating social support into self-care approaches to health behavior, such as CBT for women, is effective [51]. Women tend to benefit more from emotional support than men [55]. In health app literature [17], social support is considered an incentivizing element (e.g., likes from others) that encourages engagement and adherence, particularly for BCT-guided functions. Gender disparities have been observed in the effectiveness of social support elements in health apps [9].

Understanding the functions female users prioritize is crucial for tailoring mental health apps to meet their unique needs better, thereby enhancing user satisfaction and treatment outcomes, especially in the post-COVID era. Having established the groundwork, our subsequent research focuses on identifying the functions predominantly discussed by female users on Xiaohongshu. Therefore, we formulated the following research question:

RQ2: What dominant therapeutic functions do female users discuss in their Xiaohongshu posts related to mental health apps during COVID-19?

## 3. Materials and Methods

**Figure 1:**
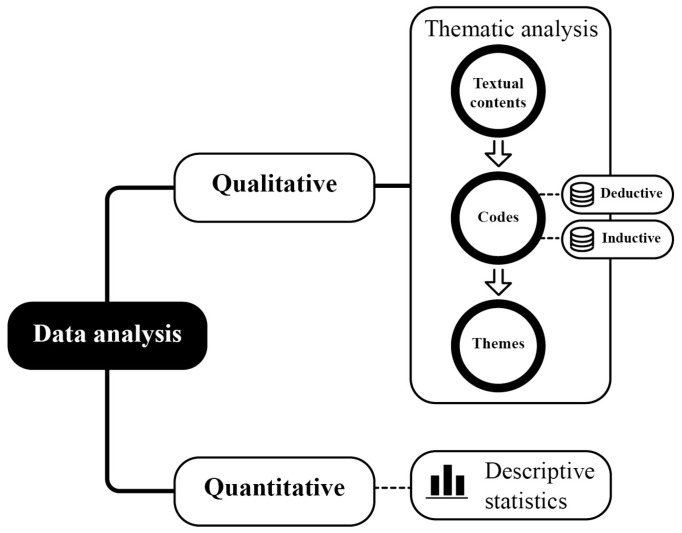
Methods of data analysis.

This study employed a mixed-methods approach. Firstly, a focused web crawler was utilized to retrieve user posts on Xiaohongshu (see details in Section 3.1). Secondly, data analysis was conducted using a mixed-methods approach encompassing qualitative thematic analysis and quantitative descriptive statistics, as depicted in Figure 1 (see details in Section 3.2). This involved post counts, which provided a quantitative overview of the data and supported our qualitative findings.

### 3.1. Data Collection

**Figure 2:**
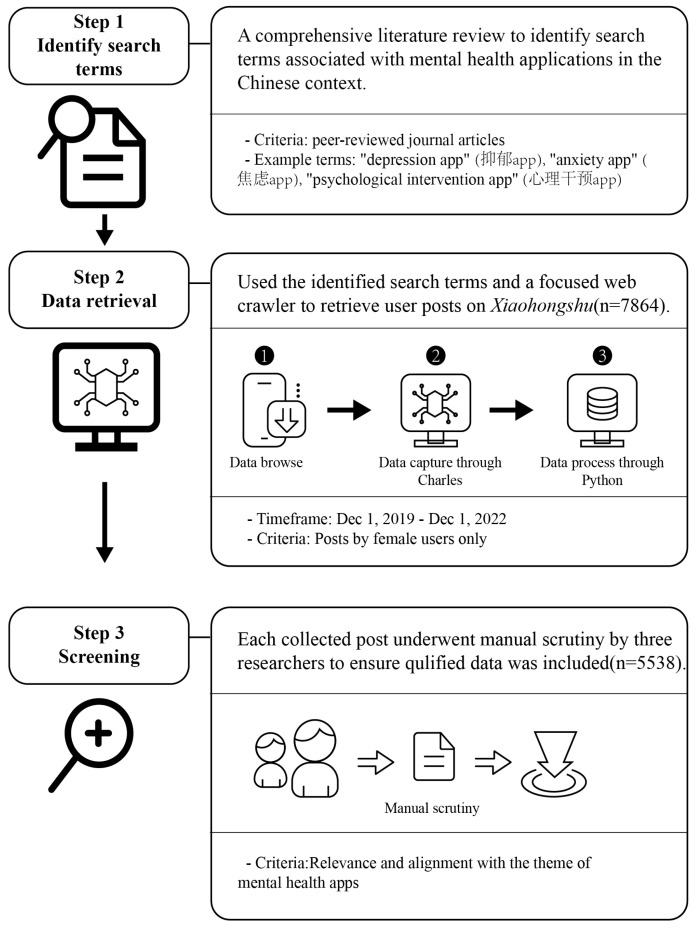
Process of data collection.

Figure 2 shows the whole process of data collection.

#### 3.1.1. Step 1: Identify Search Terms

We conducted a comprehensive literature review to identify the most pertinent and frequently used search Chinese characters associated with mental health apps. These search terms were selected based on relevant peer-reviewed studies examining online reviews of mental health apps in China [9,10,33,56,57], encompassing various categories such as “depression app”, “anxiety app”, and “psychological intervention app”.

#### 3.1.2. Step 2: Data Retrieval

We used the identified search terms and a focused web crawler to retrieve user posts on Xiaohongshu. This method was chosen as it has been demonstrated to be an effective and efficient means of searching for and retrieving resources in a specific domain [58].

The data collection process involved the following process:

- Data Browsing: Xiaohongshu pages were browsed on a mobile device to access and gather relevant data.
- Data Capture: The Charles web debugging proxy application was employed on a computer to intercept and capture the data exchanged between the mobile device and the server.
- Data Processing: The Python programming language was utilized to clean and organize the captured data, ensuring its readiness for subsequent analysis.

The focused web crawler was employed to gather posts authored by female users during the period spanning from 1 December 2019 to 1 December 2022 (n = 7864).

#### 3.1.3. Step 3: Screening

Three researchers manually scrutinized each collected post to ascertain its relevance and alignment with the theme of mental health apps. This meticulous review aimed to eliminate irrelevant or low-quality posts that could compromise our findings’ validity. Examples of such posts include those with incomplete sentences or ambiguous themes (e.g., “What’s the point of a mental health app (真不知道心理健康app有什么意义)”).

Additionally, we collected supplementary images attached to the posts to gain further insights into the perspectives of female users regarding mental health apps. Our dataset comprised 5538 textual posts, each accompanied by its corresponding date. Initially, we collected the number of likes on each post. However, since the like counts included responses to each post, making it ambiguous to count them directly and accurately, we decided not to use the number of likes in this study.

### 3.2. Data Analysis

Figure 1 illustrates the data analysis process. The study employed a mixed methods approach, combining qualitative thematic analysis and quantitative descriptive statistics. We imported all textual content and supplementary images from the collected posts into NVivo 12.0, a robust tool for qualitative data analysis [59]. We used NVivo to conduct a thematic analysis following a systematic framework [20]. This process involved several steps:

- Familiarizing with data: We thoroughly reviewed and became acquainted with the dataset.
- Defining codes: As seen in previous qualitative research [60], we employed a thematic coding process utilizing both inductive and deductive approaches. The deductive approach used existing literature to identify mental healthcare and digital service design codes based on various therapeutic theories. The inductive approach captured emerging meanings and features in the posts that did not fit within existing codes, such as those for enhanced interaction.
- Defining themes: We grouped related codes into overarching themes.
- Review of Themes: We assessed and refined the themes to ensure coherence, relevance, and alignment with the research objectives.

Furthermore, we employed descriptive statistics to detail post counts, providing a quantitative overview of the data and supporting our qualitative findings.

## 4. Results

Our analysis generated three primary themes, as shown in Table 1. Some posts involve more than one theme (n = 63). Most posts were centered on the therapeutic functions offered by mental health apps, followed by fewer posts on user experience and credibility.

### 4.1. Therapeutic Functions

Our findings suggest that female users are most concerned with the therapeutic functions of mental health apps during the COVID-19 pandemic. Based on previous literature on therapeutic functionalities of mental health apps [10,21], we classified posts into three categories of therapeutic functionality: self-care, social support, and game-based therapy. Table 2 presents the number of posts in each category.

**Figure 3:**
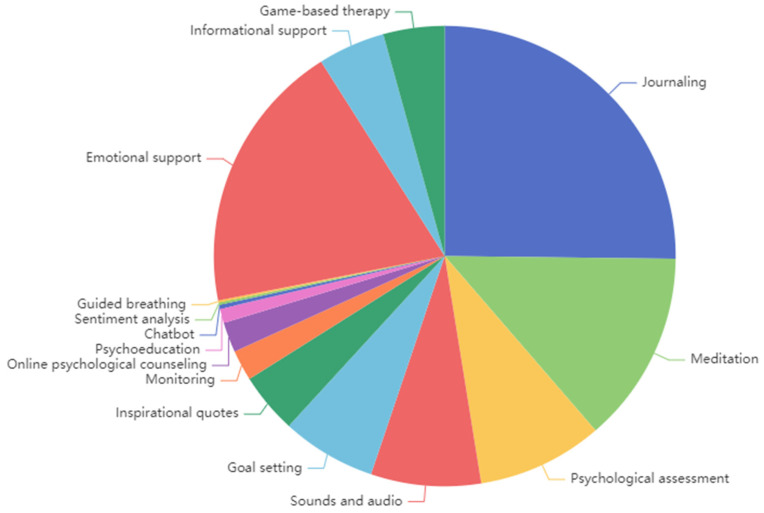
Types of therapeutic functions of mental health apps.

Figure 3 illustrates the distribution of therapeutic functions discussed in the collected posts. The analysis reveals that journaling, emotional support, meditation, and psychological assessment received the highest number of mentions during the COVID-19 pandemic. Following these were sounds and audio, goal setting, and informational support.

#### 4.1.1. Self-Care

Journaling. During the COVID-19 pandemic, female users frequently posted about mental health apps that provide well-designed journaling functions. Writing about one’s thoughts and feelings can help process and comprehend complex emotions, reducing stress and anxiety. Specific designs of journaling apps received praise from female users, with one stating, “*It is a well-designed journaling app with charming graphics that relieves anxiety. I have tried other journaling apps, but they have proven ineffective*”.

Meditation. Female users prefer guided meditation in mental health apps, especially those new to the practice, as it provides tutorials and support. Beginner-friendly meditation tools were highly recommended in collected posts, with female users commenting on their ease of use. For example, one post stated, “*This meditation tool is suitable for beginners; just follow the screen*”.

Psychological assessment. Female users have preferred psychological assessments as they provide a scientific means of understanding oneself comprehensively. For example, one user stated, “*I have suggested various assessments to my friend that can aid in self-understanding*”.

Sounds and audio. Regarding mobile technology-enhanced relaxation, female users have reported a fondness for audio-based features to overcome the negative influence of the COVID-19 pandemic, including ambient music and soundscapes, which have been lauded as “*a powerful tool for promoting relaxation*”.

Goal setting. Female users mentioned the positive impact of goal-setting functions in mental health apps. For instance, one user stated, “*I like to set a small goal every day in the app, which helps me bring about changes*”.

Inspirational quotes. Female users have discussed the benefits of mental health apps that provide daily inspirational quotes, which can give individuals the energy and motivation to overcome challenges independently. As one user noted, ‘*Whenever I need encouragement to continue living my life and maintain a positive mindset, the app throws the perfect quote for the situation at the perfect moment*’.

Monitoring. Female users frequently share their experiences with mental health apps’ monitoring functions, which enable them to track their psychological status (including mood, feelings, and thoughts), physical behaviors, and symptoms. For example, female users have shared posts such as “*The app helps me to identify when and where I feel anxious after recovering*”.

Online psychological counseling. Female users have reported that professional counseling provided online, through either text-based or audio-based communication, is a valuable and convenient means of accessing mental health support. For example, one female user commented that ‘*this online psychological counseling is convenient*’.

Psychoeducation. Female users have recommended mental health apps that offer professional psychological courses to improve their mental health knowledge. For example, one user noted that a course ‘*provides insight into how mental illnesses can be prevented and treated*’.

Chatbot. Female users have suggested mental health apps that incorporate chatbots or conversational agents, especially for anxiety and depression. Chatbots are based on computer algorithms and simulate human therapists to provide therapeutic conversations [40]. For example, one user stated, “*Instead of talking to a human, I spoke with the bot, challenged my negative thoughts, and now I feel much lighter and happier*”.

Sentiment analysis. Based on AI technology, sentiment analysis analyzes the emotional tone of users’ written or spoken text to present their mental health status. For example, a female user said, “*After the analysis, my score was abnormal*”!

Guided breathing. Female users have preferred mental health apps that provide tailored graphics and sound to guide effective breathing for relaxation. For instance, female users mentioned that “*you cannot even breathe when in bad moods”* and “*one thing that I liked was that the app also gave a visual of a ball that you follow during the practice*”.

#### 4.1.2. Social Support

According to the literature review [9], social support in mental health apps can be further categorized into emotional support, which involves design features that facilitate emotional expression and connection, and informational support, which includes conversational texts and information shared by other users.

**Figure 4:**
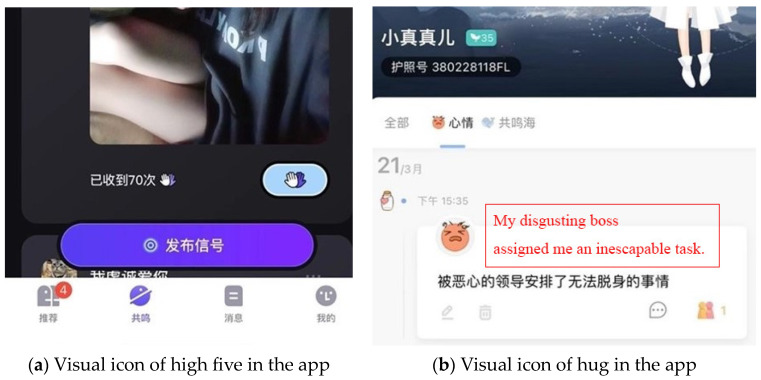
Emotional support elements found in mental health apps, identified through collected posts: (**a**) showcases a mental health app that enables users to receive emotional feedback through a clickable icon of high five in their sharing moments, with the app tallying the number of “high five” received by the user; (**b**) displays a mental health app that incorporates a clickable icon of a hug in users’ sharing moments, providing a means for users to express and receive emotional support.

Emotional support. By engaging with others who share similar emotional experiences, female users can receive empathy, encouragement, and validation, ultimately enhancing their emotional connections and feelings of social support. The inclusion of emotional support elements, such as clickable icons (e.g., liked, empathized, resonate, or hug), within mental health apps can provide virtual emotional feedback and mutual social care to users, enabling them to affirm their concerns and receive attention and a sense of identity from external sources. As an example, Figure 4 displays the high five and hugging icons in the sharing function of mental health apps, as mentioned in the collected posts.

Informational support. Interactive features in mental health apps are intended to provide instrumental information, such as comments and answers, from peers to help improve users’ mental health. Female users have reported that receiving comments within these apps has allowed them to gain a new perspective on their challenges. In particular, some users have discovered that peers who experience similar emotional states (e.g., chatting groups on specific topics) offer insightful comments that aid them in better comprehending themselves. Furthermore, hearing about the experiences of others who have overcome comparable difficulties is beneficial for some users in their mental health journey. For example, one user shared that they found it helpful when someone shared their experience of overcoming depression.

#### 4.1.3. Game-Based Interventions

**Figure 5:**
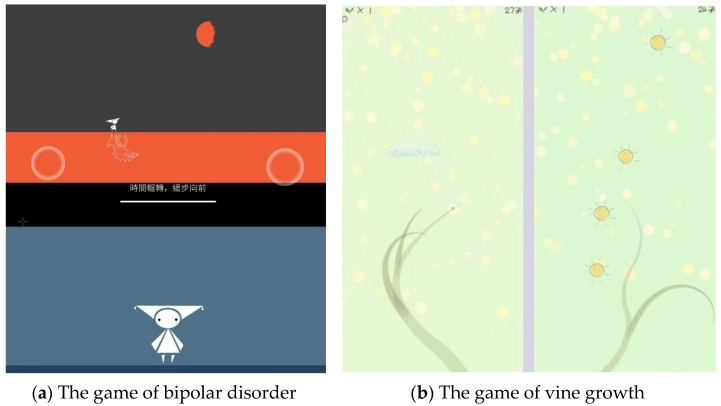
The game-based therapeutic functions featured in mental health apps from collected posts: (**a**) prompts users to engage in a gravity-balanced jumping task towards a goal, thereby replicating the psychological experiences associated with bipolar disorder; (**b**) requires players to manage the growth of a vine by utilizing gravity sensors on the touchscreen, thereby enabling the plant to absorb sunlight and flourish continually.

Female users have identified game-based features in mental health apps. These apps employ game-based mechanisms, such as rewards, challenges, goals, and progress tracking, to enhance user motivation and engagement [61]. The collected posts suggest that some mental health apps integrate game-based elements into self-care therapeutic functions, such as journaling. For instance, users can name and develop their own “mood island” and target their feelings and emotions through a mood wheel while recording life experiences. Additionally, some apps provide a game mechanism that lets users reflect on mental health issues using CBT techniques. For instance, one post described how the app’s game asks users to complete the task of gravity-balanced jumping to a goal, which mimics the psychological feelings of bipolar disorder (see Figure 5a). Other apps incorporate interactive games that offer positive psychological cues. For example, players control the growth of a vine using gravity sensors on the touchscreen, allowing the vine to absorb sunlight continuously and grow (as shown in Figure 5b). This category of apps received relatively high likes.

### 4.2. Credibility

Previous studies examining users’ credibility reviews on mental health apps [20] have highlighted vital factors for establishing credibility among female users.

#### 4.2.1. Science-Based

Female users have posted about apps that claim to have a science-backed approach, such as “*this app is developed based on the self-care theory in mental health” and “it has an evidence base*’.

#### 4.2.2. Professional Team Developed

Female users posted and liked apps that claimed to have clinical psychologists and therapists as part of their development team. For example, “*The app was developed with input from clinical psychologists*”.

### 4.3. User Experience

#### 4.3.1. Ease of Use

The collected posts from female users highlighted the significance of perceived ease of use when utilizing mental health apps. These users stressed the importance of apps that are easy to learn and operate quickly. They preferred apps with straightforward function operations, with some describing them as “*super simple*”.

#### 4.3.2. Visual Interface

Female users have emphasized the importance of visual interface design in the context of mental health apps. They recommend apps with a well-designed interface closely aligned with the app’s intended functionality. For example, one user mentioned that a mental health app with a soft green color scheme effectively conveys a sense of calm and safety. Additionally, female users have highlighted certain visual styles of interfaces, including cartoon illustrations, as particularly appealing.

#### 4.3.3. Enhanced Interaction

**Figure 6:**
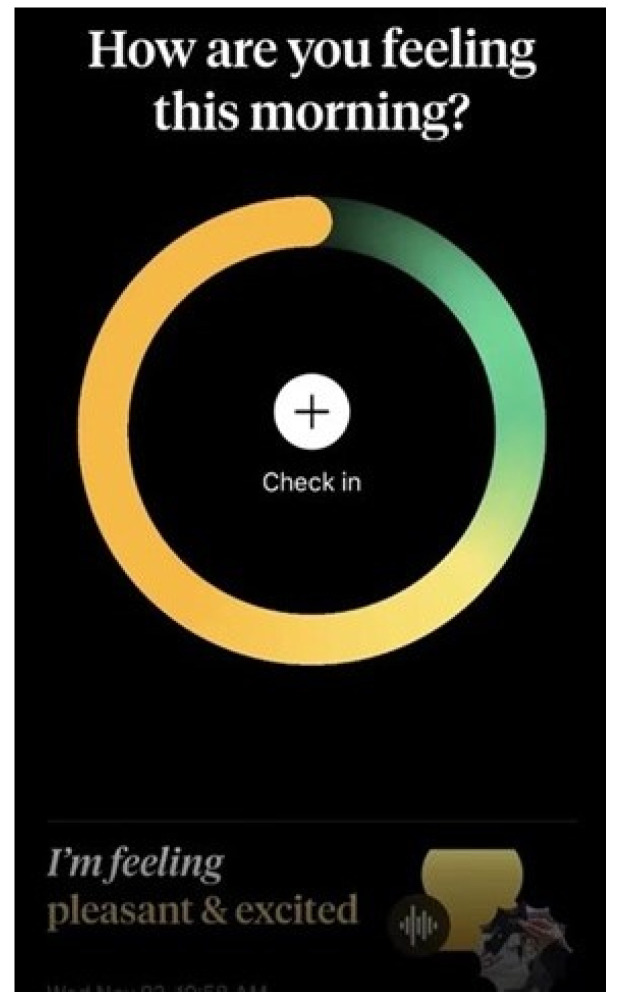
Infographic interactions in the mental health app.

Female users have preferred mental health apps that employ diverse interaction design techniques to facilitate the therapeutic process. They have highlighted the effectiveness of infographic interactions in helping them target and manage their emotions while tracking their moods within the app. This can be observed in Figure 6, which shows a screenshot of a mental health app that utilizes bipolar-colored circles to visualize the user’s emotions during mood tracking. The app uses orange to represent a pleasant mood and green to signify a calm mood, allowing the user to designate specific mood states by tapping on any location on the circle.

## 5. Discussion

Our findings align with previous research [10], suggesting the need for mental health apps to target specific adult populations, such as women. Most posts by female users were related to the various therapeutic functions of mental health apps during COVID-19. The findings suggest that self-care functionality is a crucial aspect of these apps, which aligns with the importance of self-care for mental health during the pandemic among women [47] and the recent design strategy emphasizing autonomy in aging services for female users [5]. Studies conducted during the early stages of the COVID-19 pandemic in the United States also reported the benefits of self-care behavior in addressing mental health issues [62]. This strategy aims to mobilize and empower women to improve their health conditions when hospital services are insufficient, as observed in China during the pandemic. Furthermore, Chinese female users’ preference for self-care can draw on various theoretical approaches [51,63], including BCT, CBT, WED, and the emotional approach in gender literature [64].

Among the various self-care functions of mental health apps, journaling has received the highest level of discussion from female user posts on Xiaohongshu, with the most significant posts during the pandemic. While previous research has identified journaling as a prevalent tool in mental health apps [65], it is rated relatively lower than other functions, such as monitoring and medication [20]. Lockdown policies in China during the pandemic [14] may have prompted female users to utilize disclosive tools. CBT underscores self-disclosure as a method for recognizing emotions and cognitions [66]. Scholars have also reported that self-disclosure on social media during COVID-19 in China is a mediator in enhancing well-being [67]. This preference found in our study may be linked to the observation that females tend to possess superior abilities in emotional expression and regulation [48,49]. Consequently, females may receive more benefits from emotional disclosure in mental healthcare than men [55]. This finding supports the need for tailored emotional disclosive tools for women based on CBT, such as for alcohol use disorder documented in previous work [51].

Additionally, supporting previous studies [10,20], meditation and psychological assessment are also found to be of significant interest to female users as self-care approaches. Specifically, female users have emphasized the necessity of practical guidance and support within the meditation functions of mental health apps during a pandemic. Similarly, empirical evidence indicates a positive impact of meditation on female teachers’ mental health during COVID-19 in Italy [68].

The study reveals that self-care functions essential to female users of mental health apps during the pandemic, such as inspirational quotes and goal setting, were not adequately addressed in previous research. Notably, user reviews of mental health apps have rated goal setting as a less frequently used strategy [20]. However, these features tend to enhance self-efficacy during times of uncertainty, helping to alleviate anxiety and stress caused by the pandemic. Previous evidence has shown that women experience higher levels of anxiety than men and are more likely to access self-care recommendations during COVID-19 [47]. Therefore, mental health professionals may be underrating the significance of goal setting for female users, especially during the pandemic.

In addition, inspirational quotes rank sixth in female users’ posts under the self-care category, surpassing monitoring and online psychological counseling. Facing unexpected health crises like the COVID-19 pandemic, female users may seek encouragement and confidence for the future. However, studies on using inspirational quotes in mental health apps are limited. In contrast, male patients with spina bifida and spinal cord injury were more likely to achieve goals in rehabilitation apps than females [69].

In addition to self-care, social support is an essential means of gaining energy through interactions with others [70]. Scholars [9] have reported that social support is an effective factor for increasing user engagement in health apps and that female users are more motivated by social support elements. Our study reveals that emotional and informational support are two forms of social support that can be integrated into mental health app designs. Female users value emotional support, as evidenced by the higher number of posts related to this type of support. This finding aligns with previous research emphasizing the importance of social support for women’s mental health during the COVID-19 pandemic [54]. It also aligns with the support for gender-specific CBT in women’s health treatments [51].

Our findings indicate that two factors are crucial for enhancing the credibility of mental health apps among female users. Firstly, whether the app is based on scientific evidence and whether a professional team developed it. Consistent with previous research on mental health apps, 97% of apps in China have a professional background or are evidence-based [10]. This may be related to heightened health literacy during the COVID-19 pandemic. However, our study did not find any significant posts expressing concerns about information privacy, contrasting with Alqahtani and Orji’s [20] work on the credibility of mental health apps. Our results support previous evidence emphasizing the importance of credible designs in female-centric health apps, such as pregnancy apps [35]. Compared to concerns about disclosing personal privacy, female users prioritize the credible functionality these apps provide.

Our study on the perceptions of female users of mental health apps revealed that ease of use, visual interfaces, and interactive features are critical components of their user experience. These findings are consistent with prior research on designing mental health apps for diverse audiences, which highlights the importance of interface design simplicity [23,71]. It is crucial that visual design prioritizes practicality over aesthetics and be tailored to meet the specific needs of female users, including their preferred visual styles. Additionally, incorporating various interaction design techniques can significantly enhance the overall therapeutic value of the app.

## 6. Conclusions and Design Implications

Limited research has explored the factors and potential needs of female users in mental health apps, despite evidence showing that women are more inclined to use such apps and seek designs that cater to their specific needs compared to men during the COVID-19 pandemic [9,10,29,54]. To address this gap, we collected posts from a female-centric social media platform in China to understand women’s preferences and needs regarding mental health apps during COVID-19. Our findings indicate that therapeutic functionality is crucial for female users. Also, credibility and user experience significantly influence their concerns about mental health apps. These insights, particularly relevant in the post-COVID-19 era, underscore the importance of designing gender-tailored mental health solutions that address the unique needs and preferences of female users.

Our study sheds light on the disclosure characteristics of self-care functionality among female users during the pandemic. Specifically, we found that emotional disclosure through therapeutic functions such as journaling and mood monitoring was a preferred means of emotional expression. This finding has significant implications for design researchers, highlighting the need to integrate emotional design methods such as mood granularity [72] and emotion elements [73] to enable the cognitive identification of emotions experienced by female users. Moreover, emotional design can have a semiotic capacity that elicits targeted emotional outcomes [74,75], which can lead to positive emotional changes and improve mental health outcomes. By incorporating emotional design features, designers can create more user-centric designs that cater to the emotional needs of female users based on WED and CBT-guided self-care functions.

By adopting design strategies that empower women in self-management, mental health apps can enhance prevalent self-care models like BCT-guided functions. Our study reinforces this finding, as female users highly value functions such as goal setting, which have been overlooked in previous app evaluations [20,65]. Consistent with this, giving older women more autonomy in designing healthcare services, as proposed by prior researchers [5], enables informed decision making about their health. Personalization of self-care plans to meet specific needs and preferences is a crucial approach. For example, mental health apps can offer options to adjust reminders’ frequency and types for self-care activities and track progress over time.

Incorporating social support into mental health app design is vital for promoting the mental well-being of female users during the COVID-19 pandemic. Research suggests that social interactions can create community and enhance overall wellness [76]. Designers can integrate visually emotional stimuli that facilitate mutual care, empathy, and informative feedback from peers, such as icons communicating hugs and hearts. Interactive designs can foster connections among female users with similar mental health conditions, enabling mutual emotional support and advice. These strategies build upon established social treatments like support groups in traditional health therapies.

The use of game-based mechanisms in mental health apps can be an effective way to address mental health challenges among female users. To further enhance the effectiveness of game-based interventions for female users, it may be beneficial to incorporate prevalent treatment models, including CBT, BCT, and WED principles. For instance, game-based interventions could be designed to help female users identify and modify negative thinking patterns of stigma and fear, which is a core aspect of CBT.

## 7. Limitations and Future Directions

Though this study aims to investigate a female-centric social media platform, it has several limitations. Firstly, focusing on a single social media platform within a female community may not comprehensively cover all concerns related to mental health apps. Future research should expand by including multiple platforms to gather data and conduct further studies on the specific functions and factors significant to female users, providing valuable insights for designing mental health apps.

Our study did not delve into other potentially significant aspects, such as cultural differences, socioeconomic status, or specific mental health conditions. The absence of longitudinal data is another limitation, as it could have provided insights into how user needs and preferences evolve. Future studies should address these limitations to offer a more comprehensive understanding of female users’ needs in mental health apps.

## Figures and Tables

**Table 1:** Primary themes generated in collected posts.

Theme	NoP (n = 5601)	
	n	%
Therapeutic functions	4685	83.6
User experience	682	12.2
Credibility	234	4.2

Note: NoP indicates the number of posts.

**Table 2:** A summary of therapeutic functions.

Category	Type	NoP (n = 4685)	
		n	%
Self-care	Journaling	1180	25.2
	Meditation	632	13.5
	Psychological assessment	412	8.8
	Sounds and audio	361	7.7
	Goal setting	310	6.6
	Inspirational quotes	199	4.2
	Monitoring	101	2.2
	Online psychological counseling	99	2.1
	Psychoeducation	47	1
	Chatbot	13	0.3
	Sentiment analysis	8	0.2
	Guided breathing	7	0.1
Social support	Emotional support	895	19.1
	Informational support	220	4.7
Game-based therapy		201	4.3

Note: NoP indicates the number of posts.

## Data Availability

Data are contained within the article.

## References

[1] Paganin G., Margheritti S., Farhane-Medina N.Z., Simbula S., Mazzetti G. (2023). Health, Stress, and Technologies: Integrating Technology Acceptance and Health Belief Models for Smartphone-Based Stress Intervention. Healthcare.

[2] Choudhury A., Kuehn A., Shamszare H., Shahsavar Y. (2023). Analysis of Mobile App-Based Mental Health Solutions for College Students: A Rapid Review. Healthcare.

[3] Turan N., Cetintas S. (2021). A Systematic Review of the Effectiveness, Content, and Usage Patterns of Mobile Mental Health Interventions on Smartphone Platforms for Anxiety Symptoms. J. Evid.-Based Psychother..

[4] Williams J.E., Pykett J. (2022). Mental Health Monitoring Apps for Depression and Anxiety in Children and Young People: A Scoping Review and Critical Ecological Analysis. Soc. Sci. Med..

[5] Kim M., Ramdin V., Pozzar R., Fombelle P., Zhou X., Zhang Y., Jiang M. (2022). Healthy Aging Adviser: Designing a Service to Support the Life Transitions and Autonomy of Older Adults. Des. J..

[6] Schouten S.E., Kip H., Dekkers T., Deenik J., Beerlage-de Jong N., Ludden G.D.S., Kelders S.M. (2022). Best-Practices for Co-Design Processes Involving People with Severe Mental Illness for EMental Health Interventions: A Qualitative Multi-Method Approach. Des. Health.

[7] Escoffery C. (2018). Gender Similarities and Differences for E-Health Behaviors among U.S. Adults. Telemed. E-Health.

[8] Kern A., Hong V., Song J., Lipson S.K., Eisenberg D. (2018). Mental Health Apps in a College Setting: Openness, Usage, and Attitudes. mHealth.

[9] Yin Q., Li L., Yan Z., Guo C. (2022). Understanding the Effects of Self-Peer-Platform Incentives on Users’ Physical Activity in Mobile Fitness Apps: The Role of Gender. Inf. Technol. People.

[10] Yin H., Wardenaar K.J., Wang Y., Wang N., Chen W., Zhang Y., Xu G., Schoevers R.A. (2020). Mobile Mental Health Apps in China: Systematic App Store Search. J. Med. Internet Res..

[11] Moreno C., Wykes T., Galderisi S., Nordentoft M., Crossley N., Jones N., Cannon M., Correll C.U., Byrne L., Carr S. (2020). How Mental Health Care Should Change as a Consequence of the COVID-19 Pandemic. Lancet Psychiatry.

[12] Yue J.-L., Yan W., Sun Y.-K., Yuan K., Su S.-Z., Han Y., Ravindran A.V., Kosten T., Everall I., Davey C.G. (2020). Mental Health Services for Infectious Disease Outbreaks Including COVID-19: A Rapid Systematic Review. Psychol. Med..

[13] Hudson G., Negbenose E., Neary M., Jansli S.M., Schueller S.M., Wykes T., Jilka S. (2022). Comparing Professional and Consumer Ratings of Mental Health Apps: Mixed Methods Study. JMIR Form. Res..

[14] Lau H., Khosrawipour V., Kocbach P., Mikolajczyk A., Schubert J., Bania J., Khosrawipour T. (2020). The Positive Impact of Lockdown in Wuhan on Containing the COVID-19 Outbreak in China. J. Travel Med..

[15] Du J., Mayer G., Hummel S., Oetjen N., Gronewold N., Zafar A., Schultz J.-H. (2020). Mental Health Burden in Different Professions During the Final Stage of the COVID-19 Lockdown in China: Cross-Sectional Survey Study. J. Med. Internet Res..

[16] Hou F., Bi F., Jiao R., Luo D., Song K. (2020). Gender Differences of Depression and Anxiety among Social Media Users during the COVID-19 Outbreak in China:A Cross-Sectional Study. BMC Public Health.

[17] Wang Y., Wang Y., Greene B., Sun L. (2020). An Analysis and Evaluation of Quality and Behavioral Change Techniques among Physical Activity Apps in China. Int. J. Med. Inform..

[18] Almeida M., Shrestha A., Stojanac D., Miller L. (2020). The Impact of the COVID-19 Pandemic on Women’s Mental Health. Randstad Can..

[19] Yarrington J.S., Lasser J., Garcia D., Vargas J.H., Couto D.D., Marafon T., Craske M.G., Niles A.N. (2021). Impact of the COVID-19 Pandemic on Mental Health among 157,213 Americans. J. Affect. Disord..

[20] Alqahtani F., Orji R. (2020). Insights from User Reviews to Improve Mental Health Apps. Health Inform. J..

[21] Neary M., Schueller S.M. (2018). State of the Field of Mental Health Apps. Cogn. Behav. Pract..

[22] Ng M.M., Firth J., Minen M., Torous J. (2019). User Engagement in Mental Health Apps: A Review of Measurement, Reporting, and Validity. Psychiatr. Serv..

[23] Stawarz K., Preist C., Tallon D., Wiles N., Coyle D. (2018). User Experience of Cognitive Behavioral Therapy Apps for Depression: An Analysis of App Functionality and User Reviews. J. Med. Internet Res..

[24] Li Y., Yan X., Wang Z., Ma M., Zhang B., Jia Z. (2023). Comparison of the Users’ Attitudes Toward Cannabidiol on Social Media Platforms: Topic Modeling Study. JMIR Public Health Surveill..

[25] Guo J. (2022). The Postfeminist Entrepreneurial Self and the Platformisation of Labour: A Case Study of Yesheng Female Lifestyle Bloggers on Xiaohongshu. Glob. Media China.

[26] Liu Y., Wang W. (2022). Discipline and Resistance in the Representation of Motherhood: Postpartum Recovery Discussion on Xiaohongshu. Fem. Media Stud..

[27] Yang H., Du H.S., Wang L., Wu T. (2019). The Influence of Social Support Networks on Health Conditions via User Engagement: Gender as a Moderator. J. Electron. Commer. Res..

[28] Bao H., Lee E.W.J. (2023). Examining Theoretical Frameworks and Antecedents of Health Apps and Wearables Use: A Scoping Review. Health Commun..

[29] Borrescio-Higa F., Valenzuela P. (2021). Gender Inequality and Mental Health During the COVID-19 Pandemic. Int. J. Public Health.

[30] Amerio A., Bertuccio P., Santi F., Bianchi D., Brambilla A., Morganti A., Odone A., Costanza A., Signorelli C., Aguglia A. (2022). Gender Differences in COVID-19 Lockdown Impact on Mental Health of Undergraduate Students. Front. Psychiatry.

[31] Qu P., Zhao D., Jia P., Dang S., Shi W., Wang M., Shi J. (2021). Changes in Mental Health of Women Undergoing Assisted Reproductive Technology Treatment During the COVID-19 Pandemic Outbreak in Xi’an, China. Front. Public Health.

[32] Wang X., Markert C., Sasangohar F. (2023). Investigating Popular Mental Health Mobile Application Downloads and Activity During the COVID-19 Pandemic. Hum. Factors J. Hum. Factors Ergon. Soc..

[33] Shang J., Wei S., Jin J., Zhang P. (2019). Mental Health Apps in China: Analysis and Quality Assessment. JMIR mHealth uHealth.

[34] Anderson L.N., Womack J.J., Ledford C.J.W. (2023). Initial Development and Testing of a Measure of Credibility of Mobile Health Apps: A Clinical Study among Women Seeking Prenatal Care. Atl. J. Commun..

[35] Frid G., Bogaert K., Chen K.T. (2021). Mobile Health Apps for Pregnant Women: Systematic Search, Evaluation, and Analysis of Features. J. Med. Internet Res..

[36] Torous J., Keshavan M. (2020). COVID-19, Mobile Health and Serious Mental Illness. Schizophr. Res..

[37] Alsyouf A., Masa’deh R., Albugami M., Al-Bsheish M., Lutfi A., Alsubahi N. (2021). Risk of Fear and Anxiety in Utilising Health App Surveillance Due to COVID-19: Gender Differences Analysis. Risks.

[38] Chattu V.K., Lopes C.A., Javed S., Yaya S. (2021). Fulfilling the Promise of Digital Health Interventions (DHI) to Promote Women’s Sexual, Reproductive and Mental Health in the Aftermath of COVID-19. Reprod. Health.

[39] Alves-Oliveira P., Budhiraja T., So S., Karim R., Björling E., Cakmak M. (2022). Robot-Mediated Interventions for Youth Mental Health. Des. Health.

[40] Ahmed A., Ali N., Aziz S., Abd-alrazaq A.A., Hassan A., Khalifa M., Elhusein B., Ahmed M., Ahmed M.A.S., Househ M. (2021). A Review of Mobile Chatbot Apps for Anxiety and Depression and Their Self-Care Features. Comput. Methods Programs Biomed. Update.

[41] Lucock M., Gillard S., Adams K., Simons L., White R. (2011). Review Self-Care in Mental Health Services: A Narrative Review. Health Soc. Care Community.

[42] Milne-Ives M., Lam C., De Cock C., Van Velthoven M.H., Meinert E. (2020). Mobile Apps for Health Behavior Change in Physical Activity, Diet, Drug and Alcohol Use, and Mental Health: Systematic Review. JMIR mHealth uHealth.

[43] Fedlmeier A., Bruijnes M., Vos M.B., Lemke M., Jos J. (2022). Design for Health Finding What Fits: Explorative Self-Experimentation for Health Behaviour Change Health Behaviour Change. Des. Health.

[44] MacIsaac A., Mushquash A.R., Wekerle C. (2022). Writing Yourself Well: Dispositional Self-Reflection Moderates the Effect of a Smartphone App-Based Journaling Intervention on Psychological Wellbeing across Time. Behav. Change.

[45] Frisina P.G., Borod J.C., Lepore S.J. (2004). A Meta-Analysis of the Effects of Written Emotional Disclosure on the Health Outcomes of Clinical Populations. J. Nerv. Ment. Dis..

[46] Davis P.A., Gustafsson H., Callow N., Woodman T. (2020). Written Emotional Disclosure Can Promote Athletes’ Mental Health and Performance Readiness During the COVID-19 Pandemic. Front. Psychol..

[47] Galindo-Vázquez O., Ramírez-Orozco M., Costas-Muñiz R., Mendoza-Contreras L.A., Calderillo-Ruíz G., Meneses-García A. (2023). Symptoms of Anxiety and Depression and Self-Care Behaviors during the COVID-19 Pandemic in the General Population. Gac. Med. Mex..

[48] Else-Quest N.M., Higgins A., Allison C., Morton L.C. (2012). Gender Differences in Self-Conscious Emotional Experience: A Meta-Analysis. Psychol. Bull..

[49] Wolf A. (2000). Emotional Expression Online: Gender Differences in Emoticon Use. Cyberpsychology Behav..

[50] Hides L., Dingle G., Quinn C., Stoyanov S.R., Zelenko O., Tjondronegoro D., Johnson D., Cockshaw W., Kavanagh D.J. (2019). Efficacy and Outcomes of a Music-Based Emotion Regulation Mobile App in Distressed Young People: Randomized Controlled Trial. JMIR Mhealth Uhealth.

[51] Epstein E.E., McCrady B.S., Hallgren K.A., Cook S., Jensen N.K., Hildebrandt T. (2018). A Randomized Trial of Female-Specific Cognitive Behavior Therapy for Alcohol Dependent Women. Psychol. Addict. Behav..

[52] Reblin M., Uchino B.N. (2008). Social and Emotional Support and Its Implication for Health. Curr. Opin. Psychiatry.

[53] Giurgescu C., Penckofer S., Maurer M.C., Bryant F.B. (2006). Impact of Uncertainty, Social Support, and Prenatal Coping on the Psychological Well-Being of High-Risk Pregnant Women. Nurs. Res..

[54] Corno G., Villani D., de Montigny F., Pierce T., Bouchard S., Molgora S. (2023). The Role of Perceived Social Support on Pregnant Women’s Mental Health during the COVID-19 Pandemic. J. Reprod. Infant Psychol..

[55] Fiori K.L., Denckla C.A. (2012). Social Support and Mental Health in Middle-Aged Men and Women: A Multidimensional Approach. J. Aging Health.

[56] Zhang X., Lewis S., Firth J., Chen X., Bucci S. (2021). Digital Mental Health in China: A Systematic Review. Psychol. Med..

[57] Wu X., Xu L., Li P., Tang T., Huang C. (2022). Multipurpose Mobile Apps for Mental Health in Chinese App Stores: Content Analysis and Quality Evaluation. JMIR mHealth uHealth.

[58] Xu S., Yoon H.J., Tourassi G. (2014). A User-Oriented Web Crawler for Selectively Acquiring Online Content in e-Health Research. Bioinformatics.

[59] Vaismoradi M., Turunen H., Bondas T. (2013). Content Analysis and Thematic Analysis: Implications for Conducting a Qualitative Descriptive Study. Nurs. Health Sci..

[60] Kowalski M.C., Yoon J.K. (2022). I Love It, I’ll Never Use It: Exploring Factors of Product Attachment and Their Effects on Sustainable Product Usage Behaviors. Int. J. Des..

[61] Fleming T.M., Bavin L., Stasiak K., Hermansson-Webb E., Merry S.N., Cheek C., Lucassen M., Lau H.M., Pollmuller B., Hetrick S. (2017). Serious Games and Gamification for Mental Health: Current Status and Promising Directions. Front. Psychiatry.

[62] Disabato D.J., Aurora P., Sidney P.G., Taber J.M., Thompson C.A., Coifman K.G. (2022). Self-Care Behaviors and Affect during the Early Stages of the COVID-19 Pandemic. Health Psychol..

[63] Alqahtani F., Orji R., Riper H., Mccleary N., Witteman H., Mcgrath P. (2022). Motivation-Based Approach for Tailoring Persuasive Mental Health Applications. Behav. Inf. Technol..

[64] Qiu L., Lin H., Leung A.K., Tov W. (2012). Putting Their Best Foot Forward: Emotional Disclosure on Facebook. Cyberpsychology, Behav. Soc. Netw..

[65] Six S.G., Byrne K.A., Aly H., Harris M.W. (2022). The Effect of Mental Health App Customization on Depressive Symptoms in College Students: Randomized Controlled Trial. JMIR Ment. Health.

[66] Goldfried M.R., Burckell L.A., Eubanks-Carter C. (2003). Therapist Self-disclosure in Cognitive-behavior Therapy. J. Clin. Psychol..

[67] Amosun T.S., Jianxun C., Rufai O.H., Muhideen S., Shahani R., Shah Z., Koroma J. (2022). WeChat Usage during COVID-19 Pandemic Lockdown: The Mediating Role of Online Self-Disclosure on Quality of Friendship and Well-Being. Glob. Knowl. Mem. Commun..

[68] Matiz A., Fabbro F., Paschetto A., Cantone D., Paolone A.R., Crescentini C. (2020). Positive Impact of Mindfulness Meditation on Mental Health of Female Teachers during the COVID-19 Outbreak in Italy. Int. J. Environ. Res. Public Health.

[69] Dicianno B.E., Henderson G., Parmanto B. (2017). Design of Mobile Health Tools to Promote Goal Achievement in Self-Management Tasks. JMIR mHealth uHealth.

[70] Li Y., Liang F., Xu Q., Gu S., Wang Y., Li Y., Zeng Z. (2021). Social Support, Attachment Closeness, and Self-Esteem Affect Depression in International Students in China. Front. Psychol..

[71] Chan A.H.Y., Honey M.L.L. (2022). User Perceptions of Mobile Digital Apps for Mental Health: Acceptability and Usability—An Integrative Review. J. Psychiatr. Ment. Health Nurs..

[72] Xue H., Desmet M.P., Fokkinga F.S. (2020). Mood Granularity for Design: Introducing a Holistic Typology of 20 Mood States. Int. J. Des..

[73] Ho A.G., Siu K.W.M. (2012). Emotion Design, Emotional Design, Emotionalize Design: A Review on Their Relationships from a New Perspective. Des. J..

[74] Becattini N., Borgianni Y., Cascini G., Rotini F. (2020). Investigating Users’ Reactions to Surprising Products. Des. Stud..

[75] Lin M.H., Cheng S.H. (2014). Examining the &quot;Later Wow&quot; through Operating a Metaphorical Product. Int. J. Des..

[76] Huang K.H., Deng Y.S. (2008). Social Interaction Design in Cultural Context: A Case Study of a Traditional Social Activity. Int. J. Des..

